# Unravelling biocomplexity of electroactive biofilms for producing hydrogen from biomass

**DOI:** 10.1111/1751-7915.12756

**Published:** 2017-07-11

**Authors:** Alex J. Lewis, Maria F. Campa, Terry C. Hazen, Abhijeet P. Borole

**Affiliations:** ^1^ The University of Tennessee Knoxville TN 37996 USA; ^2^ Biosciences Division Oak Ridge National Laboratory Oak Ridge TN 37831‐6226 USA; ^3^ Bredesen Center for Interdisciplinary Research and Education The University of Tennessee Knoxville TN 37996 USA; ^4^ Institute for Secure and Sustainable Environments The University of Tennessee Knoxville TN 37996 USA

## Abstract

Leveraging nature's biocomplexity for solving human problems requires better understanding of the syntrophic relationships in engineered microbiomes developed in bioreactor systems. Understanding the interactions between microbial players within the community will be key to enhancing conversion and production rates from biomass streams. Here we investigate a bioelectrochemical system employing an enriched microbial consortium for conversion of a switchgrass‐derived bio‐oil aqueous phase (BOAP) into hydrogen via microbial electrolysis (MEC). MECs offer the potential to produce hydrogen in an integrated fashion in biorefinery platforms and as a means of energy storage through decentralized production to supply hydrogen to fuelling stations, as the world strives to move towards cleaner fuels and electricity‐mediated transportation. A unique approach combining differential substrate and redox conditions revealed efficient but rate‐limiting fermentation of the compounds within BOAP by the anode microbial community through a division of labour strategy combined with multiple levels of syntrophy. Despite the fermentation limitation, the adapted abilities of the microbial community resulted in a high hydrogen productivity of 9.35 L per L‐day. Using pure acetic acid as the substrate instead of the biomass‐derived stream resulted in a three‐fold improvement in productivity. This high rate of exoelectrogenesis signifies the potential commercial feasibility of MEC technology for integration in biorefineries.

## Introduction

Many conversion technologies that could comprise the future bio‐economy are still under development, and rapid progress is needed in order to meet the growing need for renewable and carbon‐neutral energy sources. Renewable hydrogen supply and water management are among the important issues facing sustainable development of biorefineries, due to the high hydrogen demand for deoxygenation (Jones *et al*., 2013) and potential for water limitations in areas with intensive agriculture. Additionally, hydrogen in and of itself is being pursued as a renewable fuel source due to the significant reductions in tailpipe emissions that are possible via fuel cell technologies and can also serve as an energy storage mechanism for off‐peak power (FCTO, 2016). Hydrogen production from renewable sources such as biomass, however, has been lagging (Rahman *et al*., 2016). Strategies such as dark fermentation have made progress but experience low yields and carbon losses to side products and can struggle with more complex streams, while photofermentation poses operational and design challenges (Singh *et al*., 2015). Bioelectrochemical systems offer a novel way to solve these problems by recruiting biocatalysis and electrocatalysis for efficient conversion of complex biomass resources (Borole, 2011, 2015, 2016).

Engineering model organisms to convert biomass into usable bioenergy products via synthetic biology can be challenging (Cardinale and Arkin, 2012; Zuroff and Curtis, 2012), due to the complex nature of lignocellulosic biomass and the large spectrum of compounds that result from hydrolytic or thermochemical depolymerization (Kumar *et al*., 2009). The complexity of nature can be harvested to develop efficient conversion systems for energy production by repurposing the biology to solve specific human needs. In natural anoxic environments, microbial communities have evolved to degrade biomass and recycle the energy present in complex organic carbon through interactions of two main factions: fermentative and respiring bacteria. The fermentative organisms break down larger carbon compounds resulting in end‐products that are utilized by respiring bacteria to reduce nitrate, sulfate, iron or solid metals, storing the energy in cellular biomass or reduced inorganic end‐products (Lovley, 1993; Nealson and Saffarini, 1994). Bioelectrochemical systems provide a controlled environment where these processes continue to take place, but couple the electron transfer to a solid electrode, providing a means to harvest the energy as electrons and subsequently as hydrogen or other products.

While recent studies have expanded the understanding of anode microbial communities using simple fermentable substrates or domestic wastewater (Lalaurette *et al*., 2009; Parameswaran *et al*., 2009; Miceli *et al*., 2014; Mahmoud *et al*., 2016), few studies have focused on investigating the biocomplexity of engineered BESs utilizing more complex, biomass‐derived streams. There have been several studies utilizing biomass‐derived streams such as fermentation effluent or other agro/industrial waste, but have separated the fermentation from the MEC and did not focus on developing a mixed microbial community combining the fermentation and exoelectrogenesis steps (Lalaurette *et al*., 2009; Lu *et al*., 2009; Wang *et al*., 2011; Marone *et al*., 2017). Mahmoud *et al*. (2016) demonstrated the limitation of fermentation in treating more recalcitrant streams like raw landfill leachate directly in the MEC, requiring Fenton oxidation to improve biodegradability to enhance performance. Additional agro‐wastes like molasses and hydrolysates such as those from straw and corn stover conversion have been investigated directly in MECs (Thygesen *et al*., 2011; Wang *et al*., 2014; Shen *et al*., 2016). Of these, only Thygesen *et al*. tracked compound levels with time and were able to identify microbial roles for xylan degradation and propionate and acetate production, but observed low performance. Additionally, recent studies using intermediates and end‐products generated during fermentation such as carboxylic acids and alcohols have investigated their role as substrates in bioanode. Use of propionate as a substrate in MEC has revealed that it goes through a two‐step process to produce current. Hari *et al*. (2016) have delineated the pathways of propionate conversion in MEC and reported that it is first transformed into acetate and formate/hydrogen, followed by exoelectrogenesis to produce current. Similarly for butyrate, acetate has been reported to serve as a primary branching point for uptake by exoelectrogens (Miceli *et al*., 2014). Lastly, Parameswaran *et al*. (2009) demonstrated that using ethanol as the substrate, three interacting groups including fermentative bacteria, H_2_‐scavenging bacteria and exoelectrogenic bacteria were needed for successful conversion of the substrate into electrons.

Conversion of an aqueous fraction of biomass‐derived pyrolysate to electrons was recently demonstrated in a bioanode with high efficiency and productivity for renewable hydrogen production via microbial electrolysis cell (MEC; Lewis *et al*., 2015; Lewis and Borole, 2016). In order to reach levels required for commercial consideration, unravelling the biocomplexity of such a system will be key to unlocking the potential of MEC technology (Ghimire *et al*., 2015). This can support conversion of the billion‐ton biomass to biofuels and hydrogen. Thus far, studies in the literature investigating complex streams have been lacking in biocatalyst development and community interrogation. The first step in this process is to understand the multistep conversion process and the interactions among various functional groups to enable complete degradation. In order to accomplish this, an integrated approach utilizing shifts in electrochemical and substrate conditions and time‐course metabolite tracking are needed to provide insights into the resulting interactions that develop for conversion of complex substrates. Delineating the bioelectrochemical interactions and influence of process conditions on community composition can help establish the relationship between biocomplexity and system performance including yield, efficiency and rate of production of the desired products.

In this study, we report on the interaction between multiple microbial groups including fermentative and exoelectrogenic groups within a high‐performing anode community processing switchgrass‐derived bio‐oil aqueous phase (BOAP). Experiments were conducted to study the behaviour of the bioanode community under two different control regimes, one focused on changing the substrate from a complex feedstock to a substrate ideal for exoelectrogens and the other on changing the poised potential. The ideal substrate was acetic acid, which is an intermediate generated from the complex substrate BOAP, thus interrelating the two parameters. The following coupled investigations were conducted to parse the effects of the interacting parameters:

1. Conversion of BOAP under poised conditions,
2. Conversion of acetic acid under poised conditions
3. Conversion of BOAP under open‐circuit conditions to assess fermentative conversion, while restricting exoelectrogenesis

The underlying hypothesis we investigate is that the formation of acetic acid from the complex BOAP substrate is rate limiting. Sequential operation of MEC at poised and open‐circuit (unpoised) conditions provides insights into the rate at which the carbon from BOAP is directed to intermediates for exoelectrogenesis such as acetic acid and subsequently into current. Hydrogen productivity and current density as well as efficiencies of the anode, cathode and hydrogen recovery were determined. Lastly, microbial community characterization was conducted to gain insights into the relative changes in fermentative, methanogenic and exoelectrogenic populations during these experiments to understand biocomplexity.

## Results and discussion

### H_2_ Production from BOAP versus Acetic Acid in MEC

**Figure 1:**
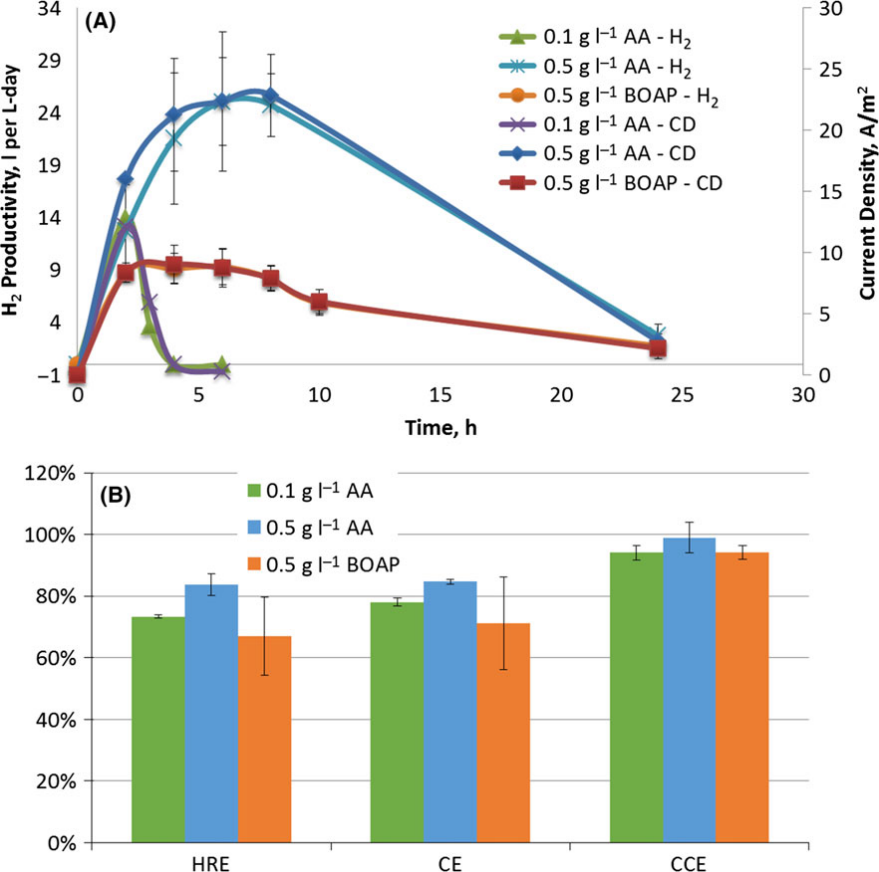
(A) Hydrogen productivity and current density for batch experiments with BOAP and acetic acid (AA) as the substrate during first 24 h. (B) Efficiency during batch experiments with BOAP and acetic acid as substrate during first 24 h.

Current and hydrogen production from two different substrates, BOAP and acetic acid was investigated to understand the transformation of BOAP in a bioanode. Acetic acid was chosen as a second substrate for investigation because this is a known intermediate for exoelectrogens and a common end‐product of fermentation reactions, although not the only one. A comparison of the current production from the two substrates has potential to reveal the relative rates of fermentation versus exoelectrogenesis in the MEC. While BOAP experiments were extended for 72 h, the results from BOAP for the first 24 h are also compared as acetic acid experiments did not run beyond this time. Total hydrogen productivity from BOAP at a concentration of 0.5 (g Chemical Oxygen Demand (COD)) l^−1^ was 4.44 ± 0.68 L per L‐day over the first 24 h. Over the same period, the hydrogen productivity using 0.5 (g COD) l^−1^ acetate was 9.05 ± 0.71 L per L‐day. At this concentration, the maximum H_2_ production rate and current density for BOAP were 9.35 ± 1.73 L per L‐day and 8.76 ± 1.54 A m^−2^ respectively (Fig. 1). In comparison, the maximum productivity and current density reached a higher peak for 0.1 (g COD) l^−1^ acetic acid. They were 1.4‐ and 1.3‐fold higher than that of BOAP, reaching 13.33 ± 0.96 L per L‐day and 11.48 ± 2.94 A m^−2^ respectively. The overall amount of H_2_ produced over the entire run was three‐fold higher with 0.5 (g COD) l^−1^ BOAP compared with 0.1 (g COD) l^−1^ acetic acid (Additional File 1: Table S2). This is not unexpected, as the BOAP experiment was fed with five‐fold more total COD. A comparison of the experiments with 0.5 (g COD) l^−1^ acetic acid and 0.5 (g COD) l^−1^ BOAP (Fig. 1) shows that the maximum H_2_ productivity and current density increased to 2.9‐fold and 2.8‐fold, reaching 27.6 ± 5.29 L per L‐day and 24.7 ± 3.64 A m^−2^ respectively. The cumulative H_2_ production from acetic acid over the duration of the experiment was 1.9‐fold higher compared with BOAP.

Looking at efficiencies during the first 24 h, BOAP produced lower hydrogen recovery (HRE), anode coulombic efficiency (CE) and cathode conversion efficiency (CCE). The anode CE, HRE and CCE for BOAP were 71.22 ± 15.08%, 66.90 ± 12.69% and 94.16 ± 2.12% respectively. For 0.1 and 0.5 (g COD) l^−1^ acetic acid, CE improved by 6.8 and 13.4% while HRE increased by 6.5 and 16.9% respectively. However, as mentioned above, BOAP conversion was slower than for acetic acid, so experimental run time was continued beyond 24–72 h. This increased overall CE by 17.5% to 88.77% ± 2.7% (Additional File 1: Fig. S2). However, HRE and CCE were reduced to 62.81 ± 9.84% and 70.96 ± 13.25% respectively. The improvement in CE for BOAP with extended run time is discussed in subsequent sections and could be result of intracellular uptake/storage during the first 24 h of BOAP conversion. The reduction in CCE and HRE for BOAP is likely the main reason for a lower total volume of H_2_ compared with 0.5 (g COD) l^−1^ acetic acid, despite similar anode efficiencies. This outcome results from a lower and more prolonged current output from BOAP, resulting in a lower average cell voltage for the run with BOAP, which reduces the efficiency of H_2_ production at the cathode. A similar observation has been reported in the literature (Gil‐Carrera *et al*., 2013; Lewis and Borole, 2016). The differences in current production from the two substrates, BOAP and acetic acid at the same concentration (0.5 (g COD) l^−1^) indicate that the bioanode community was limited by fermentation of BOAP. Second, the observation that 0.5 (g COD) l^−1^ BOAP could produce three‐fold more H_2_ compared with 0.1 (g COD) l^−1^ acetic acid at high anode efficiency indicates that the microbial community is capable of breaking down BOAP into intermediates, which serve as substrates for exoelectrogens. Another potential cause for lower current output with BOAP may be perceived to be inhibition by toxic furanic and phenolic compounds present in BOAP. However, our previous work in collaboration with Georgia Institute of Technology using the same microbial inoculum has shown that inhibition by these individual compounds and mixture of these compounds begins to occur only at a concentration two orders of magnitude higher than those used in this study (Zeng *et al*., 2015, 2016). Thus, it is unlikely that inhibition is playing a significant role in limiting the BOAP conversion. Further evidence is provided in subsequent sections. These results provide the first evidence that fermentative processes in conversion of biomass‐derived liquids may be the limiting step in this process.

Comparing hydrogen productivity and coulombic efficiency with those in the literature, Wang *et al*. were able to achieve a hydrogen production rate of 2.27 L per L‐day and CE of 95% using molasses wastewater in a single chamber MEC. Lu *et al*. (2009) reached a hydrogen production rate of 1.41 L per L‐day with a CE of 80% with fermentation effluent, which was further improved to 87% using lower applied voltage. Additionally, Li *et al*., 2014 achieved a production rate of 3.43 L per L‐day by coupling to a first step of dark fermentation to produce VFAs and reached 72% CE (Li *et al*., 2014). So despite the fermentation limitation identified in this study, the maximum productivities and efficiencies reached of 9.3 L per L‐day and 88.7% using the more recalcitrant BOAP stream compared with fermentation effluents and molasses wastewater. This demonstrates that fermentation step need not be separated from the exoelectrogenic step and that higher performance and efficiency can be achieved in a single MEC using a specifically enriched biocatalyst.

### BOAP intermediates generated during open‐circuit stimulus

**Figure 2:**
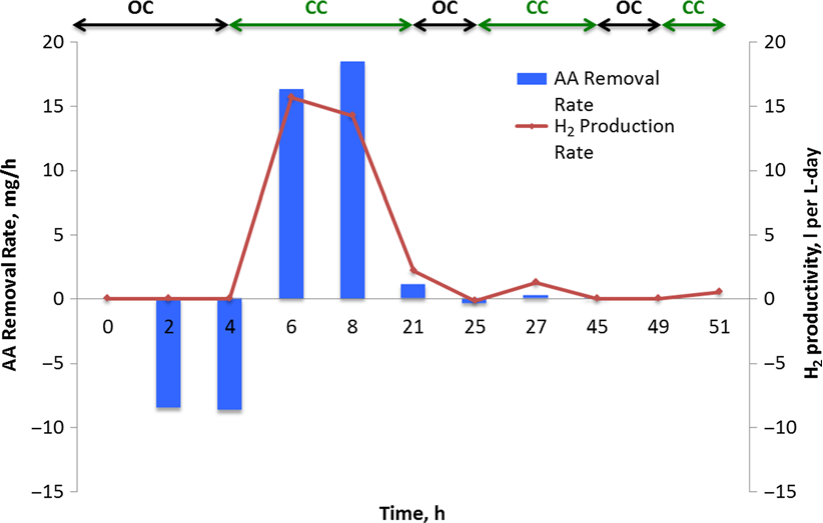
Acetic acid (AA) removal rates and hydrogen productivity during anode potential interruption experiment. OC: open‐circuit voltage, CC: set anode potential of −0.2 V versus Ag/AgCl reference electrode.

In order to determine and quantify the intermediates generated during fermentation of BOAP and to further test the hypothesis of fermentative limitations, another experiment with 0.5 (g COD) l^−1^ BOAP was carried out utilizing an open‐circuit stimulus‐response. This condition allows the system to reach open‐circuit voltage, preventing the carbon felt from acting as an electron acceptor, which halts exoelectrogenesis while enabling fermentation to proceed. During the interruption from 0 to 4 h, acetic acid accumulation was observed at a steady rate of 8.63 ± 0.13 mg h^−1^ (Fig. 2). The rate of acetic acid production may be slightly underestimated as part of it may be simultaneously consumed by exoelectrogens which have the ability to store charge (Freguia *et al*., 2007). To assess the efficiency of acetic acid production from the compounds identified by HPLC, an electron equivalence analysis was conducted as described in the Experimental section. Approximately 43.20% of the electron equivalents present in the substrate were converted to acetic acid during the first two hours of open‐circuit stimulus, which increased to 68.3% by the end of 4 h. These results demonstrate that acetic acid is the major collective fermentation end‐product from the community during the conversion of BOAP. The remaining electrons not recovered at the end of 4 h in the aqueous effluent were likely taken up by the cells to form cellular biomass or stored internally as polyhydroxyalkanoates or intracellular metabolites. The electrochemical data collected after 4 h were evidence for the latter as the coulombic efficiency obtained after poising the electrode was > 100%. Analysis of the aqueous effluent by HPLC showed that although additional fermentation by‐products were present, they were generated during closed‐circuit experiments as well. Only acetate showed the trend representative of an exoelectrogenesis substrate via heavy accumulation during open‐circuit condition, and fast removal once repoised, further suggesting that the other intermediates are not dominant fermentation end‐products in our system. Additionally, their concentration was an order of magnitude lower than acetic acid, indicating that acetic acid was the dominant branching point to exoelectrogenesis. While it is possible that some of these compounds could serve as substrates for unknown exoelectrogens, many fermentation intermediates such as propionate, butyrate, ethanol and butanol have been shown to be unsuitable for direct exoelectrogenesis (Kiely *et al*., 2011; Miceli *et al*., 2014; Hari *et al*., 2016). Additionally, as described in the community analysis section, some *β‐Proteobacteria* were found to persist during pure acetic acid experiments and thus could be diverting a small portion of acetic acid during open‐circuit stimulus.

Considering the acetic acid production rate of 8.6 mg h^−1^ during open‐circuit conditions compared with the acetic acid removal rate of 58.7 mg h^−1^ achieved using 0.5 (g COD) l^−1^ of pure acetic acid (Additional File 1: Table S3), it is clear that the exoelectrogenic microbial subpopulation is capable of converting acetic acid at higher rates than it is being produced from BOAP. Furthermore, after the circuit was closed following open‐circuit stimulus, the current production reached a higher level than what was achieved without any interruption of circuit, further indicating the exoelectrogenic subpopulation is capable of higher current output and that compounds within BOAP were not inhibitory to either of the subpopulation. Some intermediates produced from BOAP such as formate and lactate are likely substrates for exoelectrogenesis; however, they were not dominant products during the open‐circuit condition. The rate of exoelectrogenesis would be higher if they also serve as substrates for exoelectrogenesis. The observed results during open‐circuit stimulus compared with the closed‐circuit experiment thus demonstrate that the rate of fermentation was the limiting step in conversion of BOAP to current.

### Biotransformation of individual BOAP substrates under poised conditions

**Figure 3:**
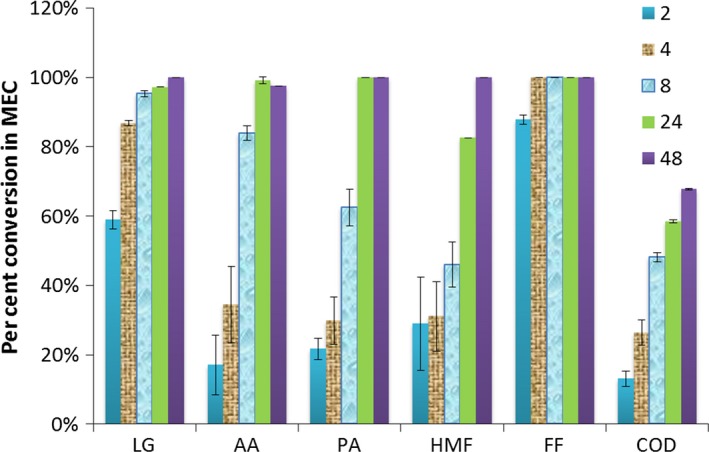
Per cent removal of individual model compounds within BOAP as measured by HPLC. The legend refers to the hours at which samples were collected.

While previous work has demonstrated significant removal of the main compounds within BOAP by the end of the run and at high efficiency (Lewis *et al*., 2015), the relative rates and order of conversion over time of the various components of the complex mixture BOAP have not been reported previously. The implications of this are significant, as pure microbial cultures can struggle with many of the lignin‐derived compounds present in BOAP (Jarboe *et al*., 2011), while microbial communities can convert complex biomass streams containing these compounds via emerging synergistic capabilities within the consortium. The composition of BOAP is outlined in Table S4. The main compounds within BOAP were all transformed simultaneously within 48 h, although at different rates (Fig. 3). Overall COD removal reached 58.4% by 24 h, and further increased to 74.8% by 72 h. For the fermentable substrates, levoglucosan had the highest initial removal rate of 16.59 ± 0.59 mg h^−1^ over the first 2 h of the batch run, followed by furfural with a rate of 1.35 ± 0.23 mg h^−1^ (Table 1). However, relative to starting concentrations, furfural had the highest initial removal percentage of 87.74 ± 1.33%, with levoglucosan reaching 58.93 ± 2.63%. 5‐hydroxymethylfurfural is another major fermentable compound present in BOAP, which was utilized at a lower rate initially with 28.88 ± 13.47% removal after two hours, but increased to 54.49 ± 6.46% after 10 h. This may be due to lower microbial density or intrinsic reaction rates. For the fermentation by‐products acetic acid and propionic acid, their initial removal rates were 6.55 ± 4.36 and 2.83 ± 0.80 mg h^−1^ respectively. However, because acetic acid is being produced through fermentation of the other compounds within BOAP simultaneously, its true removal rate is underestimated. Nevertheless, the observation that concentration of acetic acid never increased with time demonstrates that its removal outpaced production. This can also be the case for additional intermediate compounds produced during the conversion of BOAP as phenol and catechol have been identified intermediates from larger phenolic compounds in the literature (Zeng *et al*., 2017) and their concentrations were found to fluctuate during our experiments. However, their concentration at the end of the experiment was lower than the starting concentration, indicating that they were still utilized by the anode consortium.

**Table 1:** Removal rates of individual compound in mg h^−1^ during batch experiment with BOAP as the substrate during 2 h blocks of time for the first 10 h, followed by the following 14 h block

Time	2	4	6	8	10	24
Levoglucosan	16.59 ± 0.59	7.83 ± 0.59	2.36 ± 0.04	0.04 ± 0.18	−0.01 ± 0.24	0.08 ± 0.06
Acetic acid	6.55 ± 4.36	6.48 ± 2.11	9.62 ± 0.85	8.38 ± 1.06	3.66 ± 1.05	0.24 ± 0.03
Propionic acid	2.83 ± 0.8	1.07 ± 0.63	2.97 ± 0.14	1.30 ± 1.37	1.45 ± 0.88	0.47 ± 0.03
HMF	0.55 ± 0.44	−0.05 ± 0.39	0.17 ± 0.28	0.14 ± 0.03	0.17 ± 0.15	0.08 ± 0.03
2(5H)‐furanone	0.39 ± 0.1	0.02 ± 0.02	0.00	0.00	0.00	0.00
Catechol	0.07 ± 0.07	0.03 ± 0.01	−0.03 ± 0.02	0.02 ± 0.01	−0.01 ± 0.03	−0.02 ± 0.02
Furfural	1.35 ± 0.23	0.19 ± 0.01	0.00	0.00	0.00	0.00
Phenol	−0.01 ± 0.13	0.04 ± 0.04	0.01 ± 0.04	0.01 ± 0.06	0.00 ± 0.04	0.01
COD	32.43	33.01	43.43	10.42	N/A	3.19

**Figure 4:**
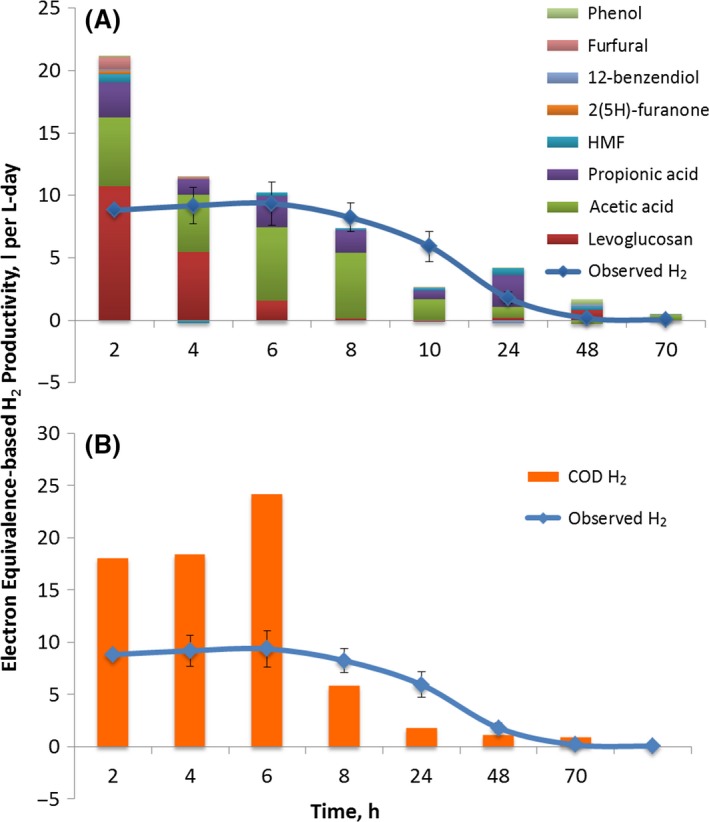
(A) Comparison of hydrogen productivity obtained experimentally with that estimated via electron equivalence calculation for conversion of individual compounds within BOAP. (B) COD contributions to hydrogen productivity based on electron equivalence compared with observed hydrogen productivity.

To further understand the productivity, efficiency and biotransformation trends observed during the conversion of BOAP, the theoretical contributions from each compound identified by HPLC towards H_2_ production were calculated via an electron equivalence calculation similar to that described in the previous section (Fig. 4). This calculation relies on the assumption that removal of the parent compounds results in their complete conversion to CO_2_, electrons and protons (Experimental Section). The bars on the *y*‐axis show equivalent rate of hydrogen production if all electrons were recovered as hydrogen at the cathode. While assuming 100% conversion is not possible, visualization in this manner allows us to estimate the extent to which the observed results deviate from this condition in discrete time frames. The results show a lag in hydrogen production compared with the rate of substrate removal. This is not unexpected as the substrate concentrations measured at the various time points are indicative of disappearance of substrate and not necessarily complete conversion. Furthermore, comparing Fig. 4A and B, we can see an inverse trend in the first 6 h, with contributions attributed to individual compound removal starting high and dropping off, while overall COD‐based contribution starts lower and increases with time. This is indicative of production of biotransformation intermediates or cellular storage, contributing to increasing COD removal from 0 to 6 h, followed by a decreasing trend thereafter. Similar to the anode electron balance described at the end of the previous section, coulombic efficiency from 6 to 24 h exceeded 100% during normal poised conditions, indicating that intracellular storage was being tapped in addition to the substrate present.

### Community analysis

**Figure 5:**
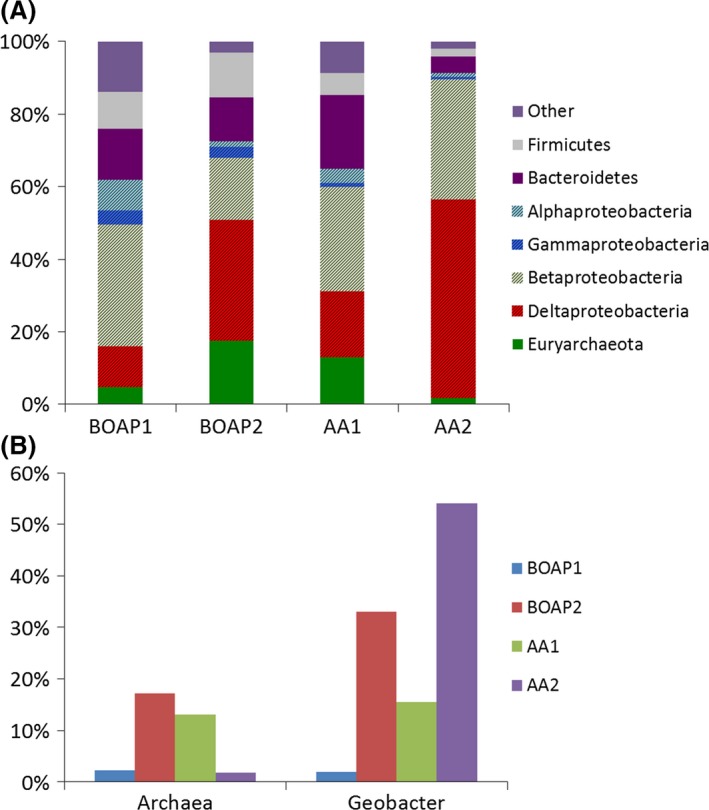
16S rRNA‐based taxonomical classification of the MEC community for batch BOAP versus acetic acid experiments. Numbers 1 and 2 indicate samples collected at the beginning (1) and the end (2) of each batch series. (A) Bar chart showing taxonomy of the MEC anode community at the phylum level with subclassification of the Proteobacteria at class level. (B) Trends in Archaea versus *Geobacter* subpopulations observed with the two substrates.

The bioanodes used in these experiments had been exposed to BOAP and adapted to this substrate for > 2 years and have evolved and enriched for BOAP conversion and acetate oxidation [6]. Focusing first on two important groups in the microbial community, exoelectrogens and methanogens, the results show different trends depending on the substrate used (Fig. 5A). Exoelectrogens, represented by the family *Geobacteraceae,* increased from 1.9% to 33.0%, when BOAP was used as the substrate. A similar trend was seen when pure acetic acid was used as the substrate, increasing the population density of the exoelectrogens from 15.6% to 54.0%. On the contrary, the population of methanogens showed an opposite trend. With BOAP as the substrate, the methanogenic *Euryarchaeota* increased from 2.2% to 17.2%, while their population decreased from 13.1% to 1.8% with acetic acid as the substrate.

Two inferences can be derived from these results. First, batch additions of acetic acid as well as BOAP provide a large amount of acetate, which is preferred by *Geobacter* for growth and exoelectrogenesis, thus explaining their growth with both substrates. Second, the fact that the population of methanogens was only observed to increase when using the complex fermentable substrate and decreased with pure acetic acid indicates the methanogens present in the anode are not acetoclastic and are likely hydrogenotrophic. Thus, the methanogen population is mainly feeding on intermediates produced during the fermentation process such as H_2_ and CO_2_ rather than the end‐product acetate, which is predominantly used by the exoelectrogenic fraction(Freguia *et al*., 2008; Ishii *et al*., 2008). The growth of methanogens is a well‐documented issue in bioelectrochemical systems even at low organic loading rates, and thus poses a significant challenge for controlling their growth at higher, industrially relevant levels (Cusick *et al*., 2011; Escapa *et al*., 2013). The difference in exoelectrogen population at the start and end of BOAP and acetic acid experiments differed greatly, which highlights the effect of substrate and fermentation limitation. Performance in terms of efficiency and current output did not improve as the *Geobacter* population increased with BOAP as the substrate during this study. This is because the amount of *Geobacter* is not the main determining factor for performance in the BOAP‐fed system, but rather a consequence of the use of BOAP in the anode. An experiment conducted after acetic acid run using BOAP as the substrate showed similar hydrogen productivity as that prior to the use of acetic acid as substrate in the MEC(Additional File, Fig. S2). As demonstrated in previous section, fermentation of BOAP to end‐products like acetate is a limiting factor in the bioanode conversion process. Once the substrate was switched to pure acetate, the exoelectrogens no longer relied on fermentation to produce the substrates they need and current production increased threefold and the *Geobacter* population increased further to 54%. Thus, limited availability of acetic acid limits *Geobacter* growth and affects its population in the anode. This was clearly illustrated by the experiment which was conducted after the acetic acid run (Additional File 1: Fig. S3). This indicates that the MEC performance is not necessarily determined by the *Geobacter* population, but by the substrate used in the MEC.

In addition to the changes in *Geobacter* and methanogen population, additional taxonomic groups including *Firmicutes*,* Bacteroidetes* and multiple classes of *Proteobacteria* also demonstrated trends as a function of substrate. *Firmicutes* and *Bacteroidetes* persisted during BOAP experiments at > 10% of the population, but were reduced significantly when acetic acid was used as the substrate. *γ‐Proteobacteria* also showed this trend but to a lower extent. This indicates that these microbes are active in the fermentation of parent or intermediate compounds from BOAP and cannot be sustained on acetate alone. *Firmicutes* have been found frequently in bioelectrochemical anodes when fermentable substrates have been used (Jung and Regan, 2007; Rismani‐Yazdi *et al*., 2007) and this phylum houses many biomass degraders and glucose fermenters in *Clostridia*. Additionally, certain microbes within *γ‐Proteobacteria* and *Bacteroidetes* have also been found to only persist when fed with fermentable sugars (Ishii *et al*., 2014). In contrast, *β‐Proteobacteria* declined during BOAP experiments but persisted during pure acetic acid, although it should be noted that this class did still remain at high levels during BOAP feeding despite the overall reduction (Fig. 5). Looking closer at the family level of *β‐Proteobacteria* (Additional File 1: Fig. S4), *Rhodocyclaceae* and *Comamonadaceae* have been identified in our previous work and house a wide metabolic range of microbes (Rismani‐Yazdi *et al*., 2007; Borole *et al*., 2009; Hesselsoe *et al*., 2009; Xing *et al*., 2010; Oren, 2014; Lewis *et al*., 2015). While these families have been implicated in degradation of complex carbon compounds, they have also been found to have abilities in acetate utilization (Ginige *et al*., 2005) and some members of *Comamonadaceae* have also been found to be capable of electricity generation (Xing *et al*., 2010), which would explain both of their abilities to persist during pure acetic acid feeding.

### Emergent functionality in engineered community

**Figure 6:**
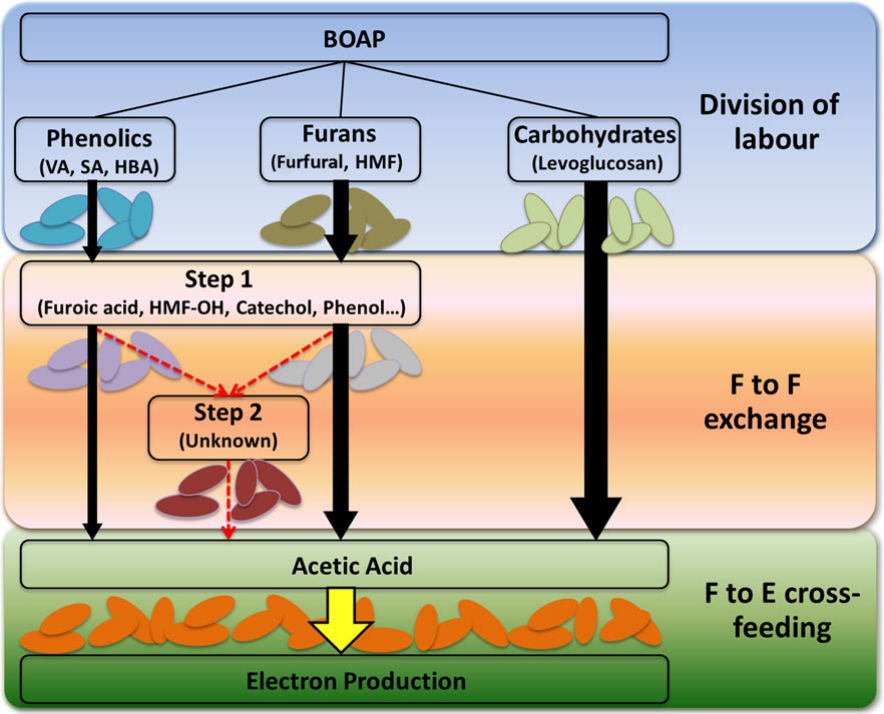
Schematic of possible pathways active in anode microbiome for conversion of fermentable compounds within BOAP. ‘F’ corresponds to fermentative bacteria, and ‘E’ corresponds to exoelectrogenic bacteria. Intermediate level 1 includes compounds such as phenol, catechol, furoic acid, which were observed experimentally. VA, vanillic acid; SA, syringic acid; HBA, hydroxybenzoic acid; HMF, hydroxymethylfurfural.

The conversion of phenolic and furanic compounds provides a significant challenge to fermentation of BOAP, as these classes of compounds, are known to be inhibitory to many microbes (Jarboe *et al*., 2011). Fermentation of the furanic compounds, furfural and HMF have been found to produce intermediates such furoic acid, furfuryl alcohol, 2,5‐bis(hydroxymethyl)furan, requiring further biotransformation to be converted to acetic acid (Wierckx *et al*., 2011; Zeng *et al*., 2015). Additionally, phenolic compounds such as phenol and catechol are even more recalcitrant, with fermentation proceeding most slowly for these types of compounds in the experiments presented in this study. Many of these intermediates including phenol, catechol and furoic acid were found in MEC effluent when BOAP served as the substrate. The comparative studies with BOAP and acetic acid show that the microbial groups were established and enriched in the anode to collectively participate in a fermentative chain that is capable of oxidizing the complex carbon compounds within BOAP progressively to acetic acid. The evidence presented in this study suggests that the microbial community utilize a synergistic strategy through mutually beneficial division of labour and syntrophic exchange as depicted in Fig. 6 that results in emergent functionality in converting the wide array of compounds within BOAP. Division of labour was evident through the simultaneous conversion of identifiable compounds, which allows for parallel processing and allocation of compounds to various microbes with different functionality and metabolic capabilities. This type of cooperative interaction within microbial communities is seen in natural environments and in engineered settings, manifesting into various ways of enhancing overall substrate utilization (Hays *et al*., 2015) (Fröstl and Overmann, 1998; Crespi, 2001; Briones and Raskin, 2003; Eiteman *et al*., 2008). This may also help prevent the toxic effects of many of these compounds on other community members. Downstream from this initial division of labour, the co‐conversions of the fermentable substrates converge to acetic acid as demonstrated by open‐circuit stimulus‐response. This leads to syntrophic cross‐feeding from fermentative groups to exoelectrogenic groups for the generation of current. This type of interaction has been demonstrated in bioelectrochemical systems before with simple substrates and is the foundation for electricity generation from fermentable substrates (Freguia *et al*., 2008; Parameswaran *et al*., 2009; Kiely *et al*., 2011). Compounds such as levoglucosan were strongly preferred within the BOAP mixture by the microbial community, which is not unexpected as it is a sugar derivative, leading to the fastest removal rate. One of the pathways through which levoglucosan can be degraded is using levoglucosan kinase to convert it to glucose 6‐phosphate (Kitamura *et al*., 1991; Zhuang and Zhang, 2002; Dai *et al*., 2009; Layton *et al*., 2011). This pathway generates acetic acid, and thus, the conversion of levoglucosan to acetic acid can occur in a single organism. Conversely, conversion of the phenolic and furanic compounds may require multiple steps. Catechol, phenol and furoic acid were found in the MEC effluent and were found to fluctuate with time during the experimental run, indicating their production from other compounds present in BOAP. These compounds have been identified as intermediates in the conversion of methoxy phenols and furanic compounds in a MEC which used the same source of enrichment used in these studies (Zeng *et al*., 2017). Thus, exchange of carbon between community members may be occurring at several levels for multiple compounds during biotransformation of the lignin and hemicellulose‐derived intermediates. Syntrophies that direct electrons away from the electrode were also evident. An increase in the population of *Euryarchaeota* which functions to redirect fermentation intermediates through methanogenesis was found. Nonetheless, CE for the BOAP batch runs reached > 80% demonstrating an efficient and robust community that can convert cellulose, hemicellulose and lignin‐derived pyrolytic intermediates including inhibitory compounds at appreciable rates, providing a foundation for further improvements to reach commercial targets.

### Bioelectrochemical systems for biorefining applications

The need for integrated solutions to the current challenges facing the world continues to grow, and new innovative approaches to fully utilize lignocellulosic feedstocks will be essential. Bioelectrochemical systems have the potential for integration into variety of bioenergy platforms to help meet this goal (Borole, 2011, 2016). MECs offer the potential to produce hydrogen in an integrated fashion in biorefinery platforms and as a means of energy storage through decentralized production to supply hydrogen to fuelling stations, as the world strives to move towards cleaner fuels and electricity‐mediated transportation. This study integrates a number of novel features into one to address practical applications such as use of MECs in biorefineries. These include:

1. An approach of comparing complex substrate, BOAP versus acetate, using open‐ and closed‐circuit conditions to understand fermentation versus exoelectrogenesis relevant to biorefinery streams
2. Community focus to understand the interactions within functional microbial groups. This can allow identifications of conditions to promote positive interactions and ways to minimize negative interactions
3. Overall integrative/systems approach, looking at metabolites (individual compounds within mixture), genomics and electrochemical data
4. Use of high‐performing MEC design capable of achieving high productivity and CE without using high concentration of substrates

The demonstrated maximum rates with BOAP in this study are nearing the targets needed for practical application (Sleutels *et al*., 2012), and almost 30‐fold higher than that identified by US DOE Fuel Cell Technology Office as state of the art (EERE, 2015). Alternate technologies such as *in vitro* synthetic enzyme systems have achieved comparatively high yields and productivities of 29 L per L‐day (54 mmol H_2_ per L‐h) utilizing both xylose and glucose from corn stover hydrolysate^44^. The study presented here is a big step towards realizing the use of renewable waste biomass as a feedstock versus sugars or natural gas for hydrogen production.

As shown in this study, developing an emergent microbial community capable of efficiently producing hydrogen from all constituents of biomass can be accomplished, but further work is necessary to increase the productivity to 20 L per L‐day or more to enable commercial consideration. This can be achieved via targeted increase in fermentative population. The generation of electrons from waste biomass has significant implications for the production of high‐value chemicals as well. This can be done via integration of electrosynthesis at the cathode (Rabaey *et al*., 2011; Borole, 2012) with the bioanode developed in this study. Future work will focus on utilizing deep sequencing techniques to better understand the interactions among active groups within the microbial community and additional environmental factors impacting performance. Building on methods previously established such as those using operational ‘shocks’ coupled to metatranscriptomic analysis identification of functional roles of different community members is possible (Ishii *et al*., 2013). Providing the right environment for improving fermentation while reducing competing pathways such as methanogenesis can enhance the production of acetic acid from BOAP. Continued efforts in these areas can lead to the development of an optimal microbial community management strategy for developing stable and high‐performing electroactive biofilms while contributing to overall strategies for engineering microbial communities for additional industrial applications.

## Conclusions

Renewable H_2_ production from biomass‐derived streams is approaching targets for practical application utilizing bioelectrochemical systems that leverage the emergent capabilities of microbial communities. A maximum productivity of 9.35 ± 1.73 L per L‐day with BOAP was achieved with a switchgrass‐derived pyrolysate. The productivity was increased threefold to 27.6 ± 5.29 l per L‐day using pure acetic acid, demonstrating the potential capability of this system. The enriched microbial community demonstrated efficient and simultaneous conversion of a wide range of compounds through synergistic division of labour strategy and multisubstrate syntrophy demonstrated by open‐circuit stimulus‐response, effectively directing the biomass electrons to intermediates such as acetic acid at an efficiency of 68.3%. However, the rate of fermentation and production of intermediates which could serve as substrates for exoelectrogenesis limited the system productivity. This study serves to provide a foundation from which to build on for understanding biocomplexity in bioelectrochemical systems for conversion of biomass‐derived streams and towards the development of community management and engineering strategies for enabling renewable hydrogen production.

## Experimental procedures

### MEC construction and experimental set‐up

Two replicate MECs were constructed with anode and cathode volumes of 16 ml each with a projected area 12.56 cm^2^. A porous carbon felt was used as anode material with a thickness of 13 and 40 mm in diameter. A membrane electrode assembly (MEA) was used as a membrane separator and cathode catalyst. The cathode consisted of Pt‐deposited carbon matching the diameter of the anode at 40 mm. Nafion 115 was used as a membrane separator between the anode and cathode chambers, and a carbon rod and stainless steel wire were used as current collectors in the two chambers respectively. Additional details of the MEC construction are reported elsewhere (Lewis *et al*., 2015).

### Bioanode enrichment

As described and characterized previously (Lewis *et al*., 2015; Lewis and Borole, 2016), bio‐oil aqueous phase (BOAP) generated from pyrolysis of switchgrass was used as substrate for enrichment of the microbial community in this study to develop a microbial community for application in biorefinery. Anode media consisted a minimal salt medium containing Wolf's mineral and vitamin solutions as reported previously. The cathode solution used was 100 mM potassium phosphate buffer.

### Batch operation

The MEC anodes utilized a flow‐through design, with the anolyte continuously recycled to and from a feed reservoir (Additional File 1: Fig. S1; Lewis *et al*., 2015). A batch concentration of 0.5 (g COD) l^−1^ was used for testing BOAP and acetic acid. Additionally, a concentration of 0.1 (g COD) l^−1^ was also used for acetic acid as this approximately corresponds to the amount of acetic acid present at time zero in 0.5 (g COD) l^−1^ BOAP. The total recirculation volume for the anode including external reservoir was 200 ml with a flow rate of 3.6 ml min^−1^. Poised conditions were maintained at −0.2 V versus Ag/AgCl via a Reference 3000 potentiostat/galvanostat/zero resistance ammeter (Gamry Instruments, Warminster PA) in all experiments. Prior to the start of each experiment, circulation of media and substrate was stopped to allow current output to decrease to the baseline level. This was done to minimize contribution of stored carbon present in biofilm cells during previous feeding. The feed reservoir was then replaced with fresh media and the circulation lines and anode chamber were flushed so that all substrate remaining from the previous experiment is removed from the whole system. The cathode buffer was not circulated and was replaced before each experiment and again after 8–10 h when pH was > 11 during the 0.5 (g COD) l^−1^ experiments. Additionally, the anode reservoir pH was adjusted at this time from 6.6 to 7.0. BOAP conversion was slower compared with acetic acid, so experimental run times were extended for BOAP experiments to 72 h, while acetic acid experiments at a concentration of 0.1 and 0.5 g l^−1^ were run for 6 and 24 h respectively. The results from first 24 h of the BOAP conversion experiment were compared with acetic acid experiments, but results for the BOAP substrate beyond 24 h are also discussed.

### Community sampling

Microbial samples were taken from the MEC anode in an anaerobic glove box utilizing a coring tool to remove a piece of the carbon felt (Lewis *et al*., 2015). Core samples were taken prior to the start of each experiment and replaced with a fresh sterile core of the same size. The core was then removed at the end of each experiment to assess how exposure to the experimental conditions impacted the composition of the community. DNA was extracted from each core using a MoBio Power Biofilm DNA extraction kit, following the manufacturer's protocol (Qiagen). Library prep was then carried out on the extracted DNA for 16S analysis on Illumina MiSeq following the methods of Caporaso *et al*. (2012). PCR products were checked via gel electrophoresis and then were pooled and run through Zymo DNA Clean and Concentrator. Samples were then checked using a Bioanalyzer and final concentration was determined by Qubit Fluorometer (Invitrogen, Carlsbad, CA, USA). Kapa qPCR was also carried out for quality control. Sequencing was carried out with Illumina MiSeq 250 bp PE run, and sequence data were analysed via qiime.

### Analysis and calculations

HPLC samples were taken every 2 h for the first 8–10 h and then at each 24 h mark thereafter. H_2_ production was measured at these times by volume displacement. At the end of each run, gas samples for GC analysis were taken from the cathode outlet to confirm hydrogen production. Liquid samples from the anode were taken from a T‐valve placed in the recirculation line prior to entering the reactor. HPLC and COD analysis were conducted to measure the extent of conversion of the substrates in BOAP. All sampling procedures were carried out as previously described (Lewis *et al*., 2015).

Performance and conversion efficiency were characterized by coulombic efficiency (CE), cathodic conversion efficiency (CCE) and hydrogen recovery (HRE) and were calculated as previously described (Logan *et al*., 2008; Lewis *et al*., 2015). Calculation of the removal rates for individual compounds and overall COD was made on the basis of each time block and was not cumulative, subtracting the mass of compound/COD measured via HPLC/COD from time point to time point. From these values, the following method was used for generating the theoretical contribution of each compound to H_2_ production:

$$\begin{array}{cc} \text{Electron}\;\text{equivalents} & =\frac{\text{concentration}\times \text{liquid}\;\text{volume}}{\text{Molecular}\;\text{weight}\;\text{of}\;\text{compound}}\times \\ & \text{moles}\;e^{-}\text{per}\;\text{mole}\;\text{of}\;\text{compound} \end{array}.$$

The electron moles for each compound are calculated from the complete oxidation of 1 mol of compound to CO_2_, protons and electrons (Additional File 1: Table S1). The electron equivalents can then be converted into theoretical volume of H_2_ through the use of two electrons per mole of H_2_ with the ideal gas law, which can then be subsequently converted to a production rate using a specified time frame within the experiment. This calculation makes the assumption that any decrease in compound concentration during batch conversion results in 100% conversion to intermediates such as acetic acid and on to electrons. To calculate the efficiency at which intermediates are produced from the compounds identified by HPLC during open‐circuit conditions, the following equation is used:

$$I_{\mathrm{eff}}=\frac{I\;\text{electron}\;\text{equiv.}}{\text{Total}\;\text{electron}\;\text{equiv.}}$$

With ‘*I*’ = intermediate, such as acetic acid, and the total electron equivalents includes all compounds that were removed during the given time frame.

**Abbreviations**
BOAP: bio‐oil aqueous phase
MEC: microbial electrolysis cell
CE: coulombic efficiency
CCE: cathodic conversion efficiency
HRE: hydrogen recovery
COD: chemical oxygen demand
AA: acetic acid
HMF: 5‐hydroxymethylfurfural
LG: levoglucosan
FF: furfural
PA: propionic acid
OC: open‐circuit
CC: closed‐circuit
VA: vanillin acid
SA: syringe acid
HBA: hydroxybenzoic acid
F: fermentor
E: exoelectrogen

## Consent for publication

Not applicable.

## Authors’ contributions

AJL designed and carried out batch experiments, electrochemical analyses, HPLC analysis, COD analysis, GC analysis, data interpretation and manuscript preparation. MFC assisted in community sample preparation and analyses. TCH provided advice on community sample analysis and assisted in manuscript preparation. APB contributed to experimental design, data analysis, interpretation and manuscript preparation and revision.

## Conflict of interest

The authors declare no competing interests.

## Supporting information


**Fig. S1.** Schematic of MEC system investigating hydrogen production under batch conditions.
**Fig. S2.** Efficiency during batch experiments with BOAP and acetic acid as substrate for entire runs.
**Fig. S3.** Hydrogen productivity during comparing run before and after acetic acid use.
**Fig. S4.** 16S r RNA‐based taxanomical classification to the family level for batch BOAP versus acetic acid. Numbers 1,2 indicate samples from beginning (1) and end (2) of each batch series.
**Table S1.** Calculation of the electrons liberated through the theoretical conversion of 1 mol of compound/COD to 1 mol of acetic acid.
**Table S2.** Cumulative hydrogen production from each batch experiment.
**Table S3.** Acetic acid and COD removal rates for each batch experiment.
**Table S4.** Concentrations of major chemical compounds in bio‐oil aqueous phase quantified by HPLC‐PDA and GC‐FID.
