# Supplementary material for: Unravelling biocomplexity of electroactive biofilms for producing hydrogen from biomass
Source: Microb Biotechnol. 2017 Jul 11;11(1):84–97. doi: 10.1111/1751-7915.12756 (PMC5743829; doi:10.1111/1751-7915.12756)
Supplement: Supplementary file 1 — Fig. S1. Schematic of MEC system investigating hydrogen production under batch conditions. Fig. S2. Efficiency during batch experiments with BOAP and acetic acid as substrate for entire runs. Fig. S3. Hydrogen productivity during comparing run before and after acetic acid use. Fig. S4. 16S r RNA‐based taxanomical classification to the family level for batch BOAP versus acetic acid. Numbers 1,2 indicate samples from beginning (1) and end (2) of each batch series. Table S1. Calculation of the electrons liberated through the theoretical conversion of 1 mol of compound/COD to 1 mol of acetic acid. Table S2. Cumulative hydrogen production from each batch experiment. Table S3. Acetic acid and COD removal rates for each batch experiment. Table S4. Concentrations of major chemical compounds in bio‐oil aqueous phase quantified by HPLC‐PDA and GC‐FID. [file MBT2-11-84-s001.pptx]

## Slide 1
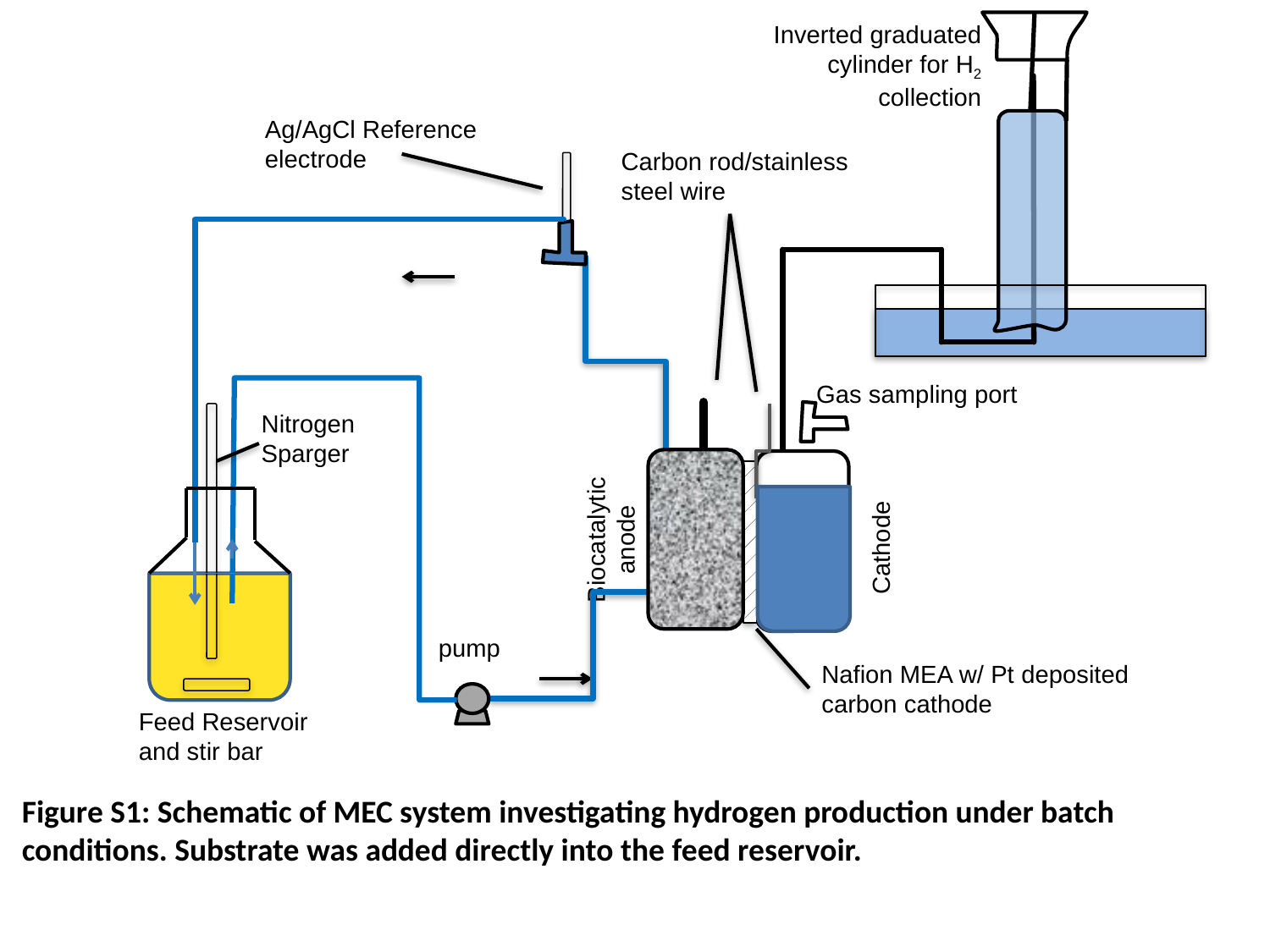

Inverted graduated cylinder for H2 collection
Ag/AgCl Reference electrode
Carbon rod/stainless steel wire
Gas sampling port
Biocatalytic anode
Cathode
pump
Nafion MEA w/ Pt deposited carbon cathode
Feed Reservoir and stir bar
Nitrogen Sparger
Figure S1: Schematic of MEC system investigating hydrogen production under batch conditions. Substrate was added directly into the feed reservoir.

## Slide 2
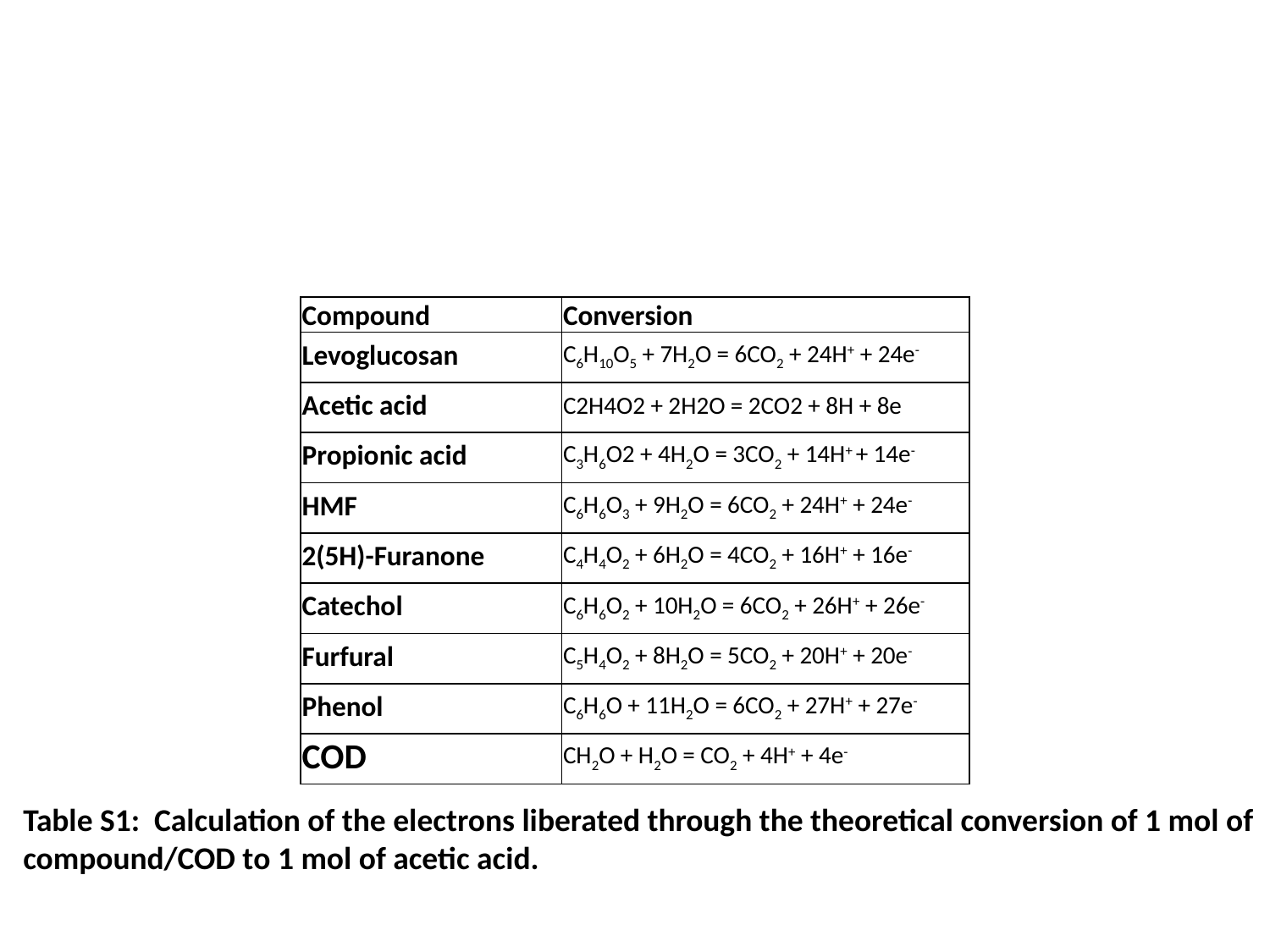

| Compound | Conversion |
| --- | --- |
| Levoglucosan | C6H10O5 + 7H2O = 6CO2 + 24H+ + 24e- |
| Acetic acid | C2H4O2 + 2H2O = 2CO2 + 8H + 8e |
| Propionic acid | C3H6O2 + 4H2O = 3CO2 + 14H+ + 14e- |
| HMF | C6H6O3 + 9H2O = 6CO2 + 24H+ + 24e- |
| 2(5H)-Furanone | C4H4O2 + 6H2O = 4CO2 + 16H+ + 16e- |
| Catechol | C6H6O2 + 10H2O = 6CO2 + 26H+ + 26e- |
| Furfural | C5H4O2 + 8H2O = 5CO2 + 20H+ + 20e- |
| Phenol | C6H6O + 11H2O = 6CO2 + 27H+ + 27e- |
| COD | CH2O + H2O = CO2 + 4H+ + 4e- |
Table S1: Calculation of the electrons liberated through the theoretical conversion of 1 mol of compound/COD to 1 mol of acetic acid.

## Slide 3
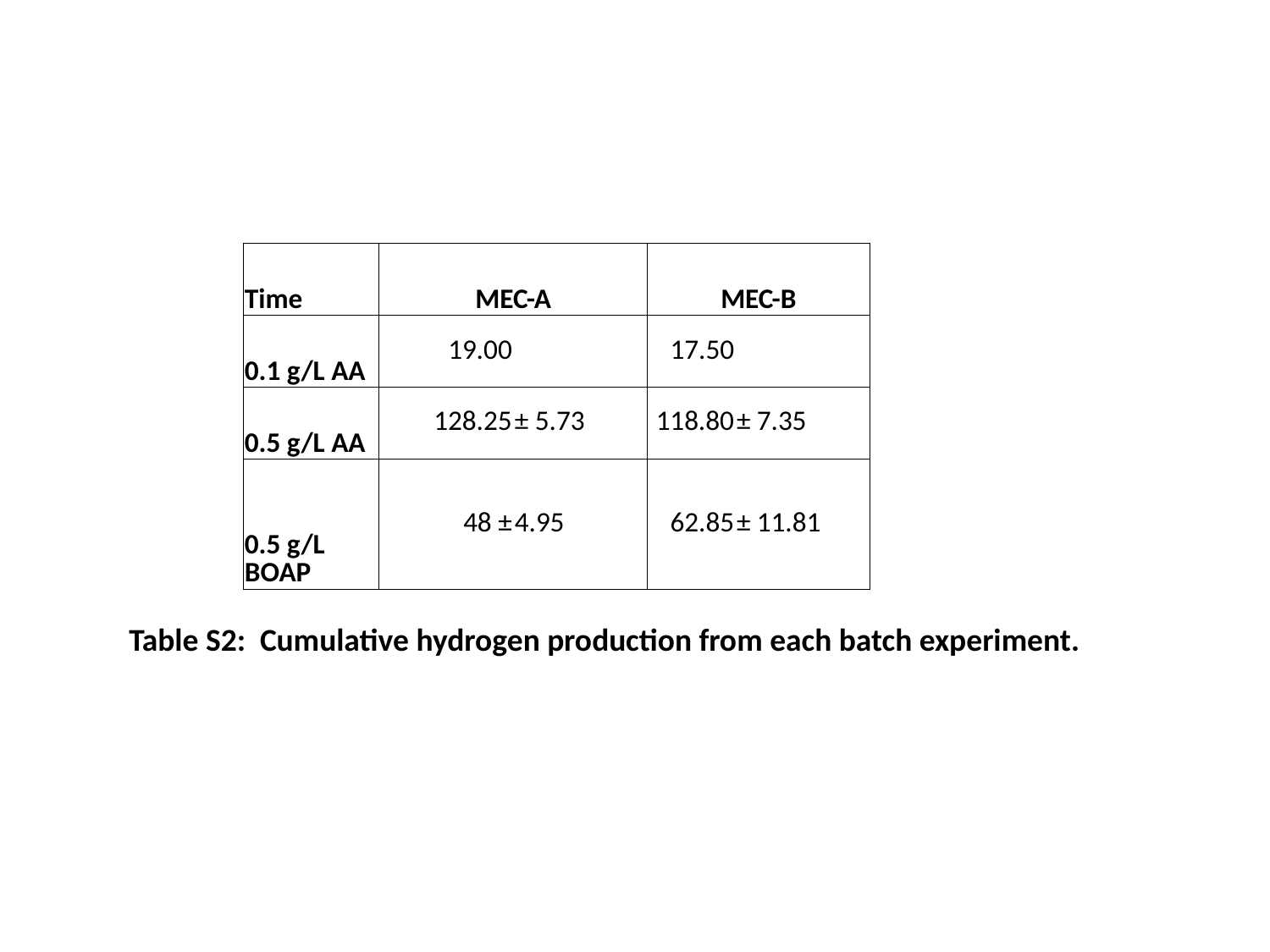

| Time | MEC-A | | MEC-B | |
| --- | --- | --- | --- | --- |
| 0.1 g/L AA | 19.00 | | 17.50 | |
| 0.5 g/L AA | 128.25 | ± 5.73 | 118.80 | ± 7.35 |
| 0.5 g/L BOAP | 48 ± | 4.95 | 62.85 | ± 11.81 |
Table S2: Cumulative hydrogen production from each batch experiment.

## Slide 4
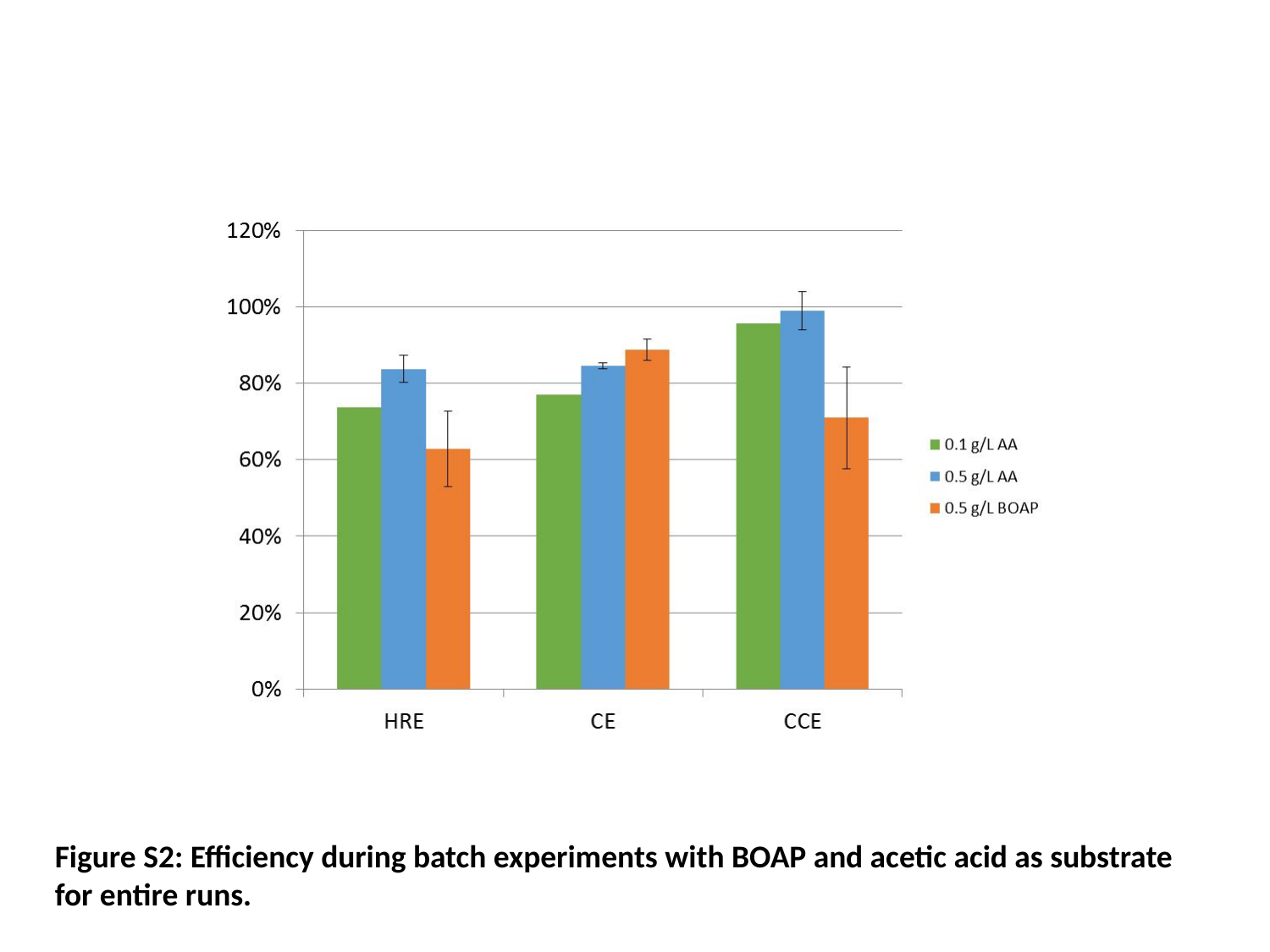

Figure S2: Efficiency during batch experiments with BOAP and acetic acid as substrate for entire runs.

## Slide 5
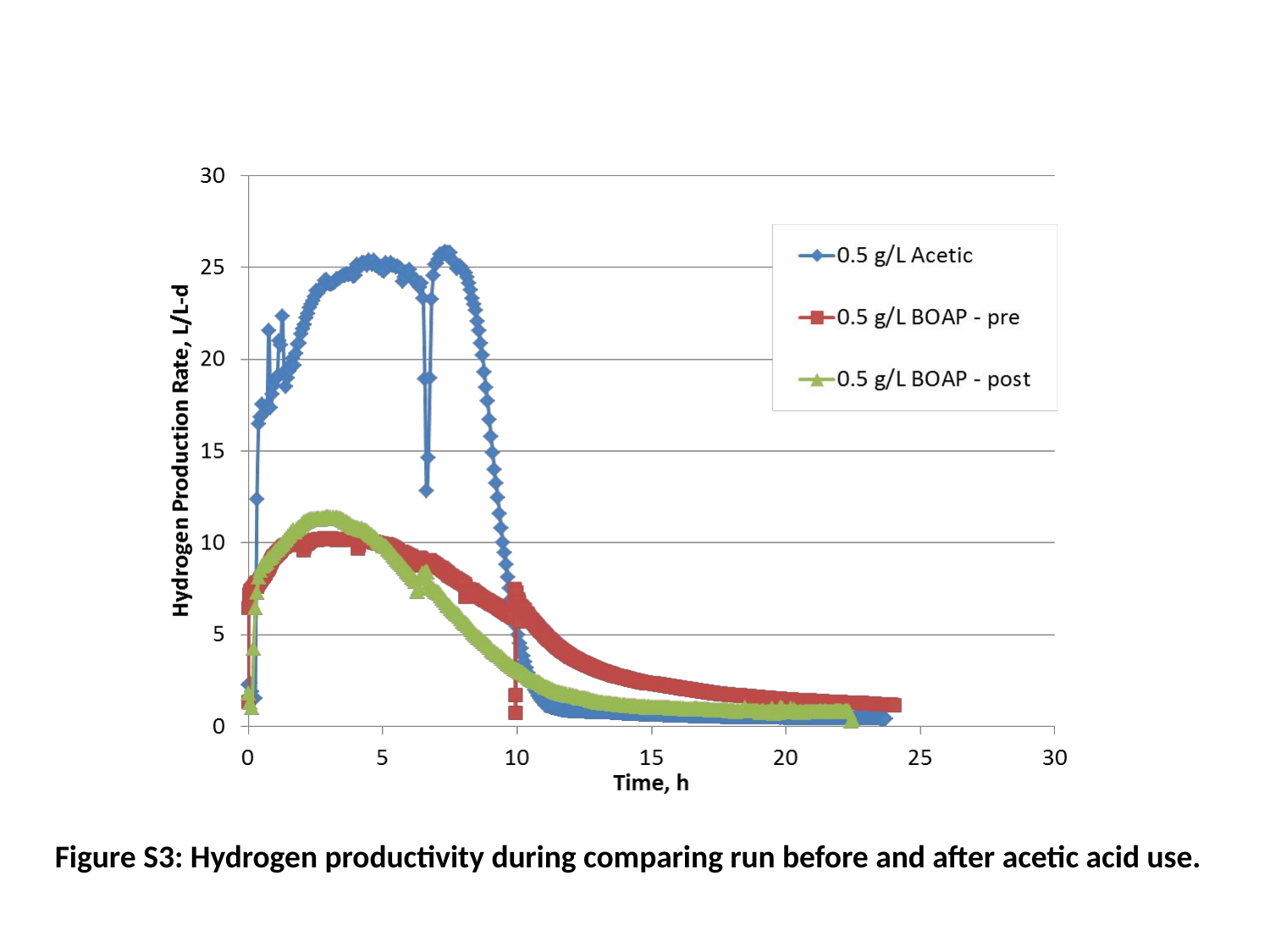

Figure S3: Hydrogen productivity during comparing run before and after acetic acid use.

## Slide 6
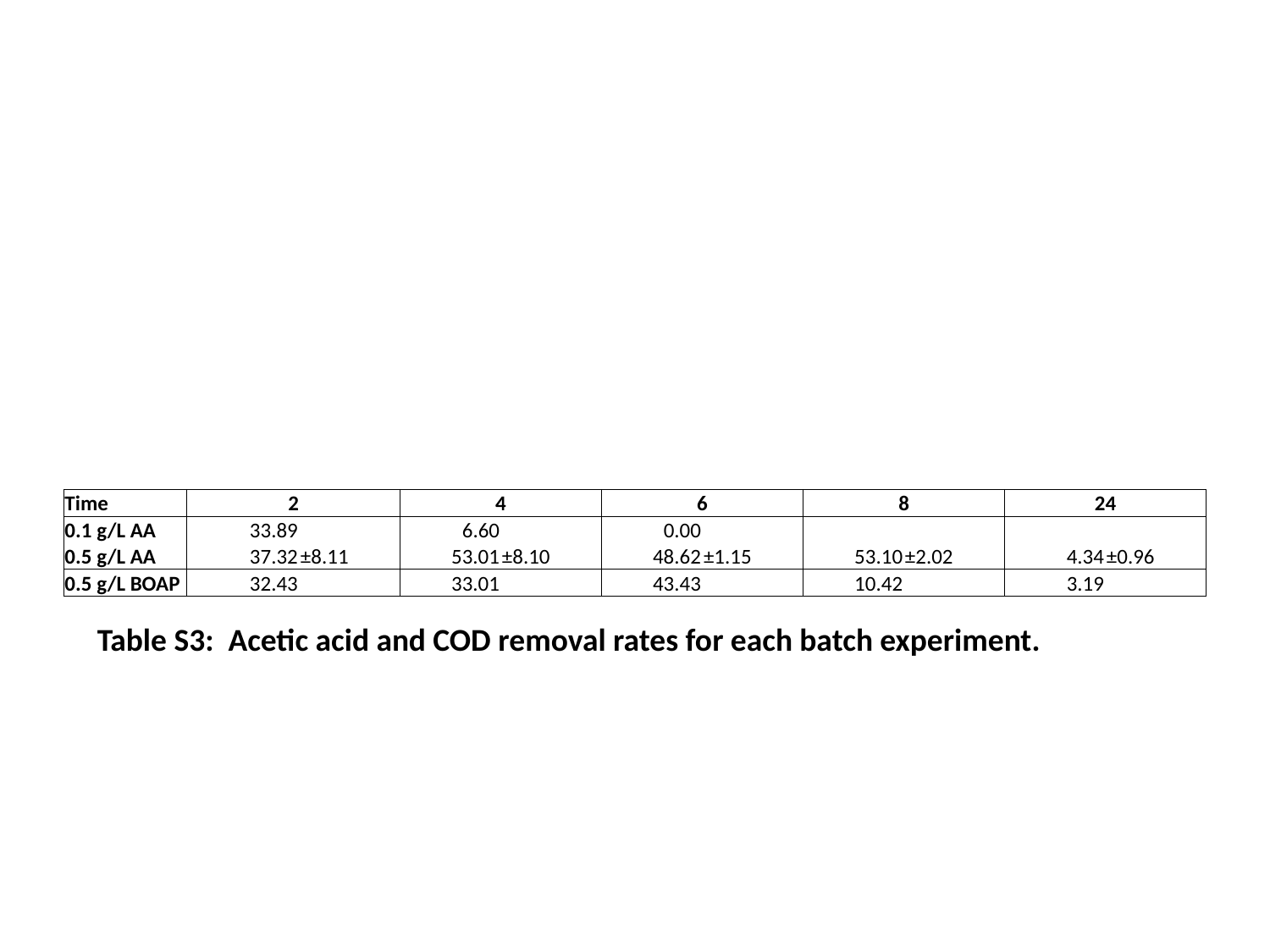

| Time | 2 | | 4 | | 6 | | 8 | | 24 | |
| --- | --- | --- | --- | --- | --- | --- | --- | --- | --- | --- |
| 0.1 g/L AA | 33.89 | | 6.60 | | 0.00 | | | | | |
| 0.5 g/L AA | 37.32 | ±8.11 | 53.01 | ±8.10 | 48.62 | ±1.15 | 53.10 | ±2.02 | 4.34 | ±0.96 |
| 0.5 g/L BOAP | 32.43 | | 33.01 | | 43.43 | | 10.42 | | 3.19 | |
Table S3: Acetic acid and COD removal rates for each batch experiment.

## Slide 7
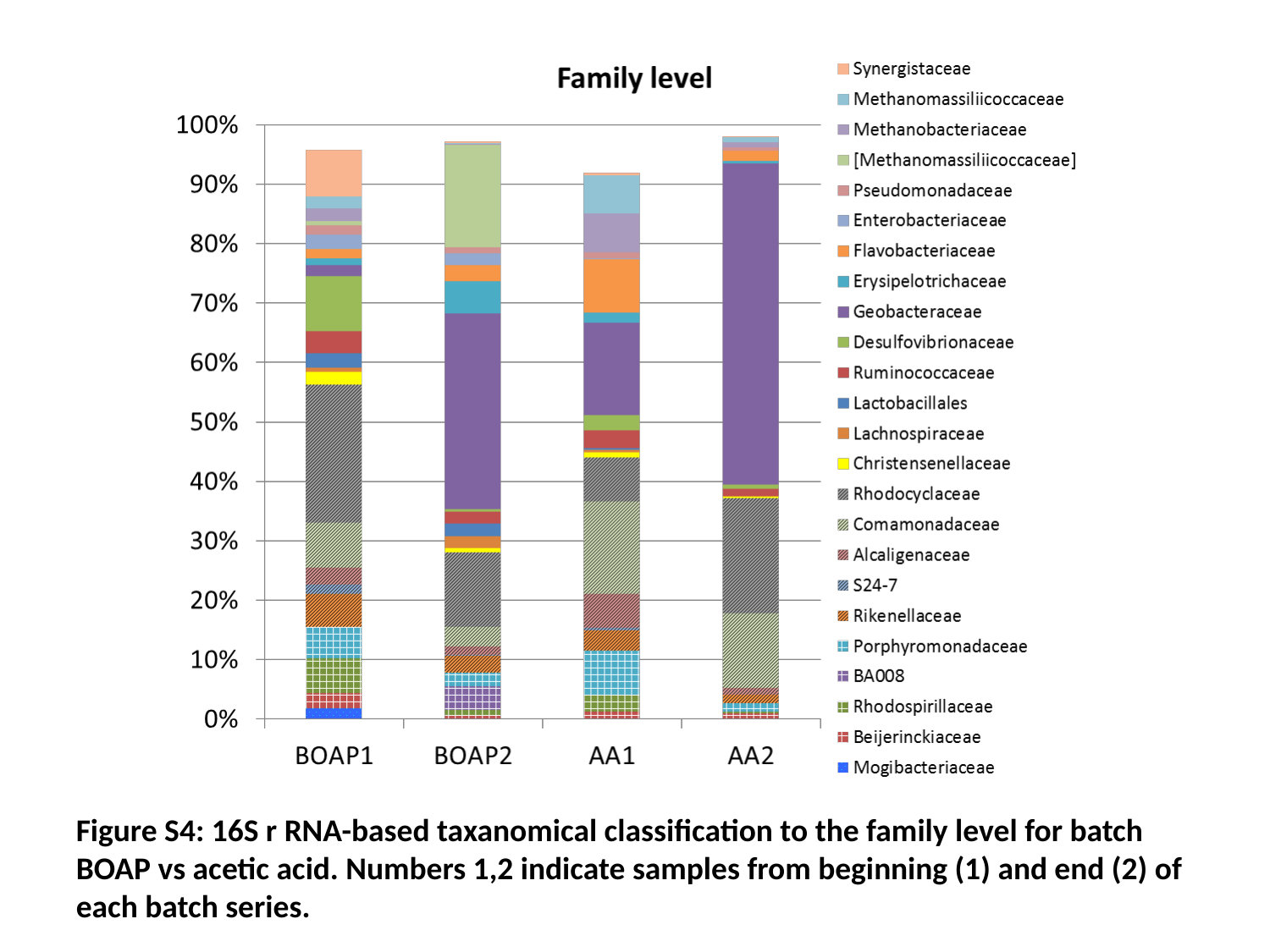

Figure S4: 16S r RNA-based taxanomical classification to the family level for batch BOAP vs acetic acid. Numbers 1,2 indicate samples from beginning (1) and end (2) of each batch series.

## Slide 8
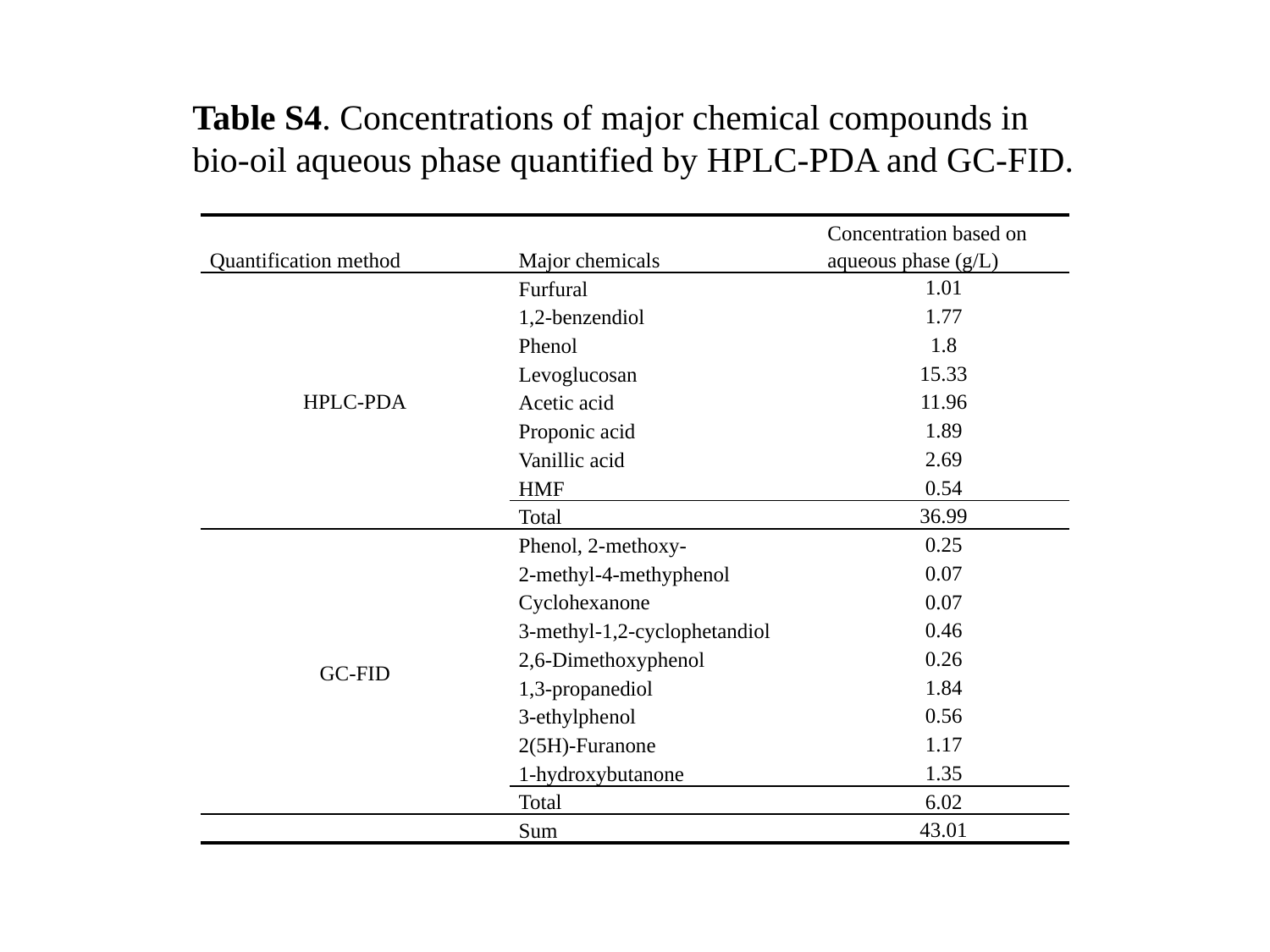

Table S4. Concentrations of major chemical compounds in bio-oil aqueous phase quantified by HPLC-PDA and GC-FID.
| Quantification method | Major chemicals | Concentration based on aqueous phase (g/L) |
| --- | --- | --- |
| HPLC-PDA | Furfural | 1.01 |
| | 1,2-benzendiol | 1.77 |
| | Phenol | 1.8 |
| | Levoglucosan | 15.33 |
| | Acetic acid | 11.96 |
| | Proponic acid | 1.89 |
| | Vanillic acid | 2.69 |
| | HMF | 0.54 |
| | Total | 36.99 |
| GC-FID | Phenol, 2-methoxy- | 0.25 |
| | 2-methyl-4-methyphenol | 0.07 |
| | Cyclohexanone | 0.07 |
| | 3-methyl-1,2-cyclophetandiol | 0.46 |
| | 2,6-Dimethoxyphenol | 0.26 |
| | 1,3-propanediol | 1.84 |
| | 3-ethylphenol | 0.56 |
| | 2(5H)-Furanone | 1.17 |
| | 1-hydroxybutanone | 1.35 |
| | Total | 6.02 |
| | Sum | 43.01 |
